# Characteristics affecting oral anticoagulant therapy choice among patients with non-valvular atrial fibrillation: a retrospective claims analysis

**DOI:** 10.1186/1472-6963-14-310

**Published:** 2014-07-17

**Authors:** Azza AbuDagga, Judith J Stephenson, An-Chen Fu, Winghan Jacqueline Kwong, Hiangkiat Tan, William S Weintraub

**Affiliations:** 1 HealthCore, Inc., 800 Delaware Ave, 5th floor, Wilmington, DE 19801, USA; 2 Daiichi Sankyo, Inc., 2 Hilton Court, Parsippany, NJ 07054, USA; 3 Christiana Care Health System, 4755 Ogletown-Stanton Road, Suite 2E99, Newark, DE 19718, USA

**Keywords:** Non-valvular atrial fibrillation, Dabigatran, Warfarin, Oral anticoagulation, Healthcare

## Abstract

**Background:**

Dabigatran is one of the three newer oral anticoagulants (OACs) recently approved in the United States for stroke prevention in non-valvular atrial fibrillation (NVAF) patients. The objective of this study was to identify patient, healthcare provider, and health plan factors associated with dabigatran versus warfarin use among NVAF patients.

**Methods:**

Administrative claims data from patients with ≥2 NVAF medical claims in the HealthCore Integrated Research Database between 10/1/2009 and 10/31/2011 were analyzed. During the study intake period (10/1/2010 - 10/31/2011), dabigatran patients had ≥2 dabigatran prescriptions, warfarin patients had ≥2 warfarin and no dabigatran prescriptions, and the first oral anticoagulant (OAC) prescription date was the index date. Continuous enrollment for 12 months preceding (“pre-index”) and ≥ 6 months following the index date was required. Patients without pre-index warfarin use were assigned to the ‘OAC-naïve’ subgroup. Separate analyses were performed for ‘all-patient’ and ‘OAC-naïve’ cohorts. Multivariable logistic regression (LR) identified factors associated with dabigatran versus warfarin use.

**Results:**

Of 20,320 patients (3,019 dabigatran and 17,301 warfarin) who met study criteria, 27% of dabigatran and 13% of warfarin patients were OAC-naïve. Among all-patients, dabigatran patients were younger (mean 67 versus 73 years, p < 0.001), predominantly male (71% versus 61%, p < 0.001), and more frequently had a cardiologist prescriber (51% versus 30%, p < 0.001) than warfarin patients. Warfarin patients had higher pre-index Elixhauser Comorbidity Index (mean: 4.3 versus 4.0, p < 0.001) and higher ATRIA bleeding risk score (mean: 3.0 versus 2.3, p < 0.001). LR results were generally consistent between all- and OAC-naïve patients. Among OAC-naïve patients, strongest factors associated with dabigatran use were prescriber specialty (OR = 3.59, 95% CI 2.68-4.81 for cardiologist; OR = 2.22, 95% CI 1.65-2.97 for other specialist), health plan type (OR = 1.47 95% CI 1.10-1.96 for preferred provider organization), and prior ischemic stroke (OR = 1.42, 95% CI 1.06-1.90). Older age decreased the probability of dabigatran use.

**Conclusions:**

Beside patient characteristics, cardiology specialty of the prescribing physician and health plan type were the strongest factors associated with dabigatran use.

## Background

Three new oral anticoagulants (OACs) [dabigatran, rivaroxaban, and apixaban] have recently been approved for stroke prevention in atrial fibrillation (AF) patients in the United States (US). These new agents have fixed dosing regimens, do not require routine monitoring of the patient’s coagulation status, and have less food-drug interaction potential than warfarin [1-3]. In a randomized clinical trial, dabigatran 150 mg was superior in stroke reduction and similar in the risk of major bleeding compared to warfarin [4]. In a double-blind randomized clinical trial, rivaroxaban was shown to have similar efficacy in stroke prevention as warfarin based on an intent-to-treat analysis, but superior efficacy and similar risk of major bleeding compared to warfarin based on a per protocol analysis [5]. Clinical trial results also indicated a significant reduction in the risk of stroke and risk of major bleeding was associated with apixaban compared to warfarin [6]. Overall, these OACs have been found to have either similar or better efficacy or safety profiles, offer more therapeutic options and may provide better outcomes for stroke prevention among AF patients compared to warfarin [4-6].

The American College of Chest Physicians (ACCP) 2012 guidelines recommend that in the absence of elevated bleeding risk or contraindications, patients at increased risk of stroke should be treated with an OAC [7]. Despite the availability and the established safety of OACs, they are underutilized among patients with non-valvular atrial fibrillation (NVAF) for whom OAC use is indicated [8]. High bleeding risk, perceived low stroke risk, brief duration of AF, and personal preference have been cited by physicians as reasons for not prescribing warfarin to patients with AF [9].

Due to the recent availability of the newer OACs, little research exists about patient, healthcare provider, and health plan factors related to their use compared to warfarin among NVAF patients in the real-world. This research is needed because patient and health plan characteristics, [10-12], along with specialty characteristic of the healthcare provider [13-15] have been shown to affect patient use of treatment and/or patient outcomes in cardiovascular and other diseases. Cost, safety and efficacy, patient preference, and cardiology specialty may be important factors affecting the choice of OAC among likely dabigatran users [16]. This study sought to identify patient, provider, and health plan characteristics associated with the use of dabigatran versus warfarin among a large cohort of NVAF patients using real-world data.

## Methods

### Study population and inclusion criteria

The study was a retrospective analysis of medical and pharmacy claims utilizing administrative claims data from the HealthCore Integrated Research Database (HIRD^SM^), a large database of administrative claims for from a large health benefits organization. The HIRD^SM^ contains longitudinal claims data for approximately 45 million patients belonging to 14 health plans in the Northeastern, Southern, Midwestern, and Western regions of the US; it includes patients covered by health maintenance organizations (HMOs), preferred provider organizations (PPOs), and other health plans (including point of service (POS), consumer-directed health, and indemnity health plans).

All study materials were handled in compliance with the Health Insurance Portability and Accountability Act (HIPAA), and a limited dataset (as defined in the Privacy Regulations issued under HIPAA) was used for this analysis. Patient confidentiality was preserved and the anonymity of all patient data was safeguarded throughout the study. Institutional Review Board approval was not required for this retrospective observational study in which researchers only had access to a limited data set that excluded specified patient identifiers and consisted of anonymized patient information.

### Patient identification

Eligible patients were identified from the HIRD^SM^ during the study period from October 1, 2009 to April 30, 2012. Patient aged 18 years or older were required to have at least two medical claims for AF as primary or secondary diagnosis (ICD-9-CM code 427.31) separated by at least 30 days between October 1, 2009 and October 31, 2011. At least one of these AF medical claims had to be an outpatient claim and both were required to occur prior to or coincide with the study index date (see below for definition). Patients were classified into dabigatran and warfarin cohorts based on their OAC prescription claims filled during the study intake period (from October 1, 2010 to October 31, 2011). During the intake period, dabigatran patients had ≥2 dabigatran prescriptions, warfarin patients had ≥2 warfarin and no dabigatran prescriptions, and the first OAC prescription fill date served as the index date. Continuous health plan enrollment for 12 months preceding (“pre-index period”) and ≥ 6 months following the index date was required. Patients with valvular heart disease/valve replacement or hyperthyroidism (determined via ICD-9-CM codes (Table 1) in the 12-month pre-index period were excluded. Patients without any prescription claims for an oral anticoagulant during the pre-index period were classified as oral anticoagulant naïve (OAC-naïve) patients. Separate analyses were performed for the ‘all-patient’ and ‘OAC-naïve’ cohorts.

**Table 1:** Patient exclusion criteria

**Condition**	**ICD-9 diagnosis codes**	**ICD-9 procedure codes**	**HCPCS codes**	**CPT codes**
Valvular disease	394.x–397.x, 398.9x,093.2x,391.1x, 398.90,424.xx, 746.0x-46.7x,996.02, 996.71	35.0x-5.2x, 35.31,35.32, 35.33,35.95-35.97, 35.99	-	33400-33496, 33600, 33602, 33660, 33665, 33670, 33684, 92986-92990
Hyperthyroidism	242	-	-	-

### Study measures of interest

The primary outcome variable was use of dabigatran or warfarin on the study index date. Independent variables of interest included patient pre-index demographic and clinical characteristics, healthcare provider specialty, and health plan factors. Demographic characteristics included patient age (at the index date) and gender. Healthcare variables included health plan geographic location (Northeast, Midwest, South, West), and type of health plan [HMO, PPO, other]. A healthcare provider factor (the index OAC prescriber specialty (cardiology, primary care/family/internal medicine,other specialty) was examined. Total patient pre-index out-of-pocket prescription drug costs were calculated.

Comorbid conditions were identified by ICD-9-CM codes associated with medical claims at any position during the pre-index period and included the following: coronary artery disease, myocardial infarction, cardiomyopathy, prior ischemic stroke, prior transient ischemic attack (TIA), prior bleed, heart failure, hypertension, peripheral artery disease, diabetes, and stages II-V chronic kidney disease. In addition, the Elixhauser comorbidity index (ECI) [17,18] was used to assess patient overall pre-index comorbidity burden, using ICD-9-CM codes for 30 conditions associated with medical claims during the pre-index period. CHADS_2_[19] stroke risk factors and total score(calculated based on presence of heart failure (HF), hypertension, diabetes, prior stroke/TIA, and age >75 years) and ATRIA [20] bleeding risk scores (calculated based on presence of anemia, severe renal disease, any prior hemorrhage diagnosis, hypertension, and age ≥75 years) were calculated for study cohorts. AF-related procedures (heart rate and heart rhythm procedures, including insertion of pacemaker or defibrillator, cardioversion, surgical ablation, and left atrial catheter ablation) during the pre-index were assessed.

Additionally, pharmacy prescriptions dispensed during the pre-index period were described for the following medications: warfarin (all-patients cohort only), parenteral anticoagulants (enoxaparin, tinzaparin, dalteparin, fondaparinux), anti-platelet therapy/NSAIDS (clopidogrel, ticagrelor, prasugrel, ticlopidine, cilostazol, dipyridamole, and non-steroidal anti-inflammatory agents [NSAIDS]), heart rate control medications (beta-blockers, calcium channel blockers, digoxin/digitoxin), antiarrhythmic medications (dronedarone, amiodarone, disopyramide, procainamide, flecainide, propafenone, sotalol, deofetilide, quinidine, mexiletine, moricizine), and dyspepsia medications (proton pump inhibitors and histamine receptor antagonists). Finally, the presence of ≥1 prothrombin time (PT) test during the pre-index period was assessed (all-patients cohort only) as a proxy for presence of ≥1 INR tests.

### Statistical analysis

Patient demographic and clinical characteristics, healthcare provider specialty, and health plan factors associated with dabigatran or warfarin use on the index date were analyzed separately for all-patient and OAC-naïve patient cohorts. Two-sample t-tests were used to test differences between continuous variables, chi-square tests were used to test differences between categorical variables, and Wilcoxon rank-sum tests were used to test differences in the cost variables between the dabigatran and warfarin cohorts.

Multivariable logistic regression (LR) with backward elimination was used to identify significant factors associated with dabigatran (reference group = warfarin) use. Separate LR models were conducted for all-patient and for OAC-naïve patient cohorts. The following independent variables were used in the LR models: patient age in years (defined as 18–54, 55–64, 65–74, 75–84, and ≥85), patient gender, ECI score, ATRIA bleeding risk score, separate indicator variables for the presence of relevant pre-index comorbidities (HF, hypertension, diabetes, ischemic stroke, transient ischemic attack, stages II-V chronic kidney disease); indicator variable for pre-index AF-related procedures; pre-index use of warfarin, pre-index PT test (only among all-patient cohort); separate indicator variables for pre-index use of parenteral anticoagulants, anti-platelet/NSAIDS therapy, heart rate control medications or antiarrhythmic medications, and dyspepsia medications; and health plan variables (health plan type and geographic region), the specialty of index OAC prescriber, and total patient pre-index out-of-pocket prescription drug costs. Female and age group variables were forced into the LR models.

## Results

### Descriptive analysis results

Overall, 20,320 patients who received either dabigatran or warfarin therapy on the index date met the study criteria (“all-patient cohort”): 14.9% (3,019) received dabigatran, while 85.1% (17,301) received warfarin. Among patients who received dabigatran, 464 (15.4%) also received warfarin prescriptions during the study follow-up. Among OAC-naïve patients (n = 2,405): 815 (33.9%) patients received dabigatran and 1,590 (66.1%) received warfarin and had no dabigatran prescriptions during the pre-index period.

Patient demographic and clinical characteristics, healthcare provider specialty, and health plan factors between dabigatran and warfarin cohorts were comparable among the all-patient and OAC-naïve cohorts (Table 2). Dabigatran patients were significantly younger than warfarin patients [all-patient cohort: mean (SD) age 67 (11.4) versus 73 (11.4) years, p < 0.0001; OAC-naïve cohort: mean (SD) age 65 (11.9) versus 71 (12.1) years, p < 0.0001] and less frequently females (all-patient: 29.1% versus 39.2%, p < 0.001; OAC-naïve: 27.5% versus 35.4%, p < 0.0001). Among all- and OAC-naïve patients, dabigatran users were more frequently covered by a preferred provider organization (PPO) benefit design than a HMO benefit design (all-patient: 67.8% versus 49.3%, p < 0.0001; OAC- naïve: 68.2% versus 52.9%, p < 0.0001). Compared to warfarin, dabigatran use was less frequent in the Midwest (all-patient: 21.5% versus 36.6%, p < 0.001; OAC-naïve: 22.7% versus 44.9%, p < 0.0001) and more frequent in the West health plans (all-patient: 32.0% versus 22.9%, p < 0.001; OAC-naïve: 32.1% versus 22.4%, p < 0.0001). Patients were more likely to be prescribed dabigatran as their index OAC by a cardiologist (rather than a primary care, or other provider) than warfarin patients (all-patient: 51.0% versus 30.3%, p < 0.001; OAC- naïve: 51.3% versus 32.7%, p < 0.001). Mean patient out-of-pocket pharmacy costs during the pre-index period was higher for dabigatran than warfarin patients [all-patient: mean (SD) $1,012 ($1,585) versus $952 ($1,050), p < 0.0001; OAC-naïve: mean (SD) $870 ($2,135) versus $655 ($735), p < 0.0001].

**Table 2:** Pre-index patient demographic, healthcare provider, and health plan characteristics

**Characteristics**	**All patient cohorts**					**OAC-naïve cohorts**				
	**Dabigatran (n = 3,019)**		**Warfarin (n = 17,301)**		**P-value**	**Dabigatran (n = 815)**		**Warfarin (n = 1,590)**		**P-value**
	**N/mean**	**%/SD**	**N/mean**	**%/SD**		**N/mean**	**%/SD**	**N/mean**	**%/SD**	
**Patient demographic variables**										
**Age (years), n,%**										
18-44	57	1.9	147	0.8	<.0001	30	3.7	22	1.4	<.0001
45-54	297	9.8	897	5.2		108	13.3	132	8.3	
55-64	1,040	34.4	3,299	19.1		313	38.4	363	22.8	
65-74	777	25.7	4,213	24.4		191	23.4	375	23.6	
75-84	619	20.5	5,969	34.5		124	15.2	486	30.6	
85+	229	7.6	2,776	16.0		49	6.0	212	13.3	
**Age, mean, SD**	67	±11.4	73	±11.4	<.0001	65	±11.9	71	±12.1	<.0001
**Female**	878	29.1	6,775	39.2	<.0001	224	27.5	563	35.4	<.0001
**Health plan and healthcare provider variables**										
**Insurance plan, n,%**										
Preferred provider organization	2,047	67.8	8,522	49.3	<.0001	556	68.2	841	52.9	<.0001
Health maintenance organization	396	13.1	4,136	23.9		108	13.3	308	19.4	
Other	576	19.1	4,643	26.8		151	18.5	441	27.7	
**Health plan region, n,%**										
Midwest	648	21.5	6,333	36.6	<.0001	185	22.7	714	44.9	<.0001
Northeast	720	23.8	4,257	24.6		175	21.5	300	18.9	
West	966	32.0	3,962	22.9		262	32.1	356	22.4	
South	685	22.7	2,749	15.9		193	23.7	220	13.8	
**Physician specialty, n,%**										
Cardiology	1,541	51.0	5,234	30.3	<.0001	418	51.3	520	32.7	<.0001
Primary care/internal medicine	424	14.1	6,132	35.5		75	9.2	418	26.3	
Other	1,054	34.9	5,935	34.2		322	39.5	652	41.0	
**Patient out of pocket pharmacy costs during the pre-index period**	$1,012	$1,585	$952	$1,050	0.0080	$870	±$2,135	$655	±$735	0.0003

Compared to warfarin patients, dabigatran patients had lower CHADS_2_ risk scores (all patients: mean (SD) 1.9 (1.3) versus 2.2 (1.3), p < 0.0001; OAC- naïve: mean (SD) 1.7 (1.4) versus 2.2 (1.4), p < 0.0001)), lower ATRIA scores (all patient: mean (SD) 2.3 (2.1) versus 3.0 (2.3), p < 0.0001; OAC- naïve: mean (SD) 2.0 (2.0) versus 3.1 (2.6), p < 0.0001)) (Table 3). Dabigatran patients also had fewer pre-index comorbidities than warfarin patients (all patient: mean (SD) ECI 4.0 (2.2) versus 4.3 (2.3), p < 0.0001; OAC-naïve: mean (SD) ECI 3.7 (2.1) versus 4.5 (2.6), p < 0.0001). Dabigatran patients more frequently had a history of ischemic stroke or TIA, but less frequently had hypertension, diabetes, and HF than warfarin users. Dabigatran patients more frequently had pre-index AF-related procedures than warfarin patients among the all-patient cohort (5.1% versus 2.5%, p < 0.0001), but there was no significant difference in the proportion of patients with pre-index stroke/TIA or AF-related procedures between OAC-naïve dabigatran and OAC-naïve warfarin patients.

**Table 3:** Patient pre-index clinical characteristics

**Characteristics**	**All patient cohorts**					**OAC-naïve cohorts**				
	**Dabigatran (n = 3,019)**		**Warfarin (n = 17,301)**		**P-value**	**Dabigatran (n = 815)**		**Warfarin (n = 1,590)**		**P-value**
	**N/mean**	**%/SD**	**N/mean**	**%/SD**		**N/mean**	**%/SD**	**N/mean**	**%/SD**	
**Summary comorbidity/severity scores**										
CHADS_2_, mean, SD	1.9	±1.3	2.2	±1.3	<.0001	1.7	±1.4	2.2	±1.4	<.0001
ATRIA, mean, SD	2.3	±2.1	3.0	±2.3	<.0001	2.0	±2.0	3.1	±2.6	<.0001
Elixhauser (ECI), mean, SD	4.0	±2.2	4.3	±2.3	<.0001	3.7	±2.1	4.5	±2.6	<.0001
**Selected pre-index comorbidities, n,%**										
Any ischemic stroke	497	16.5	2,567	14.8	0.0213	127	15.6	259	16.3	0.6550
Any TIAs	169	5.6	789	4.6	0.0131	46	5.6	90	5.7	0.9870
Heart failure	603	20.0	4,592	26.5	<.0001	136	16.7	445	28.0	<.0001
Hypertension	2,394	79.3	14,202	82.1	0.0003	626	76.8	1,288	81.0	0.0157
Diabetes mellitus	895	29.6	5,683	32.8	0.0005	207	25.4	508	31.9	0.0009
Chronic kidney disease (stages II-V)	172	5.7	1,265	7.3	0.0014	45	5.5	154	9.7	0.0005
Heart rate and heart rhythm control procedures*	155	5.1	436	2.5	<.0001	44	5.4	66	4.1	0.1656
**Pre-index use of selected medications**										
Use of warfarin, n,%	2,204	73.0	15,711	90.8	<.0001	0	0.0	0	0.0	NA
≥1 PT tests, n,%	2,111	69.9	13,575	78.5	<.0001	NA	NA	NA	NA	NA
Parenteral anticoagulants, n,%	231	7.7	974	5.6	<.0001	13	1.6	59	3.7	0.0040
Any platelet inhibitor	648	21.5	2,768	16.0	<.0001	225	27.6	341	21.4	0.0007
Prescription NSAIDs	456	15.1	1,791	10.4	<.0001	145	17.8	190	11.9	<.0001
Any rate control medication	2,475	82.0	13,916	80.4	0.0472	614	75.3	1,119	70.04	0.0103
Any rhythm control medication, n,%	948	31.4	3,238	18.7	<.0001	286	35.1	353	22.2	<.0001
Any rate or rhythm control medications	2,562	84.86	14,223	82.21	0.0004	654	80.3	1,164	73.2	0.0001
Any dyspepsia medication, n,%	749	24.8	4,122	23.8	0.2424	173	21.3	362	22.8	0.3900
Proton-pump inhibitor (PPI)	689	22.8	3,701	21.4	0.0781	159	19.5	320	20.1	0.7201
Histamine type-2 receptor antagonist (H2RA)	100	3.3	583	3.4	0.8717	26	3.2	55	3.5	0.7293

*Includes insertion of pacemaker or defibrillator, cardioversion – electrical, left atrial catheter ablation and surgical ablation.

Among the all-patient cohort, more warfarin patients used warfarin during the pre-index period compared to dabigatran patients (90.8% versus 73.0%, p < 0.0001) (Table 3). Dabigatran patients more frequently used anti-platelet agents/NSAIDS than warfarin patients during the pre-index period among both the all-patient and OAC-naïve cohorts (all-patient: 21.5% versus 16.0%, p < .00001; OAC- naïve: 27.6% versus 21.4%, p < 0.0007). Use of parenteral anticoagulants was more common among dabigatran users in the all-patient cohorts, but less frequent among dabigatran than warfarin patients in the OAC-naïve patients (all patient: 7.7% versus 5.6%, p < 0.0001; OAC-naïve: 1.6% versus 3.7%, p < 0.0040). Use of any heart rate or rhythm control medication was more common among dabigatran than warfarin patients (all-patient: 84.9% versus 82.2%, p < 0.0004; OAC-naïve: 80.2% versus 73.2%, p < 0.0001). Among the all-patient cohort, warfarin users were more likely to have ≥1 PT tests during the pre-index period (90.8% versus 73.0%, p < 0.0001).

### Multivariable LR results

The factors that were significantly associated with dabigatran use were generally similar for the all-patient and OAC-naïve patient LR analyses (Table 4); the null hypothesis for lack of model fit was rejected based on a Hosmer and Lemeshow test for the all-patients model (p-value = 0.6416) and for the OAC-naïve patients model (p-value = 0.0754), indicating that both models fit the data well. In both analyses, the adjusted odds of dabigatran use decreased with increasing patient age among patients 65 or older (range of OR = 0.39-0.64; all p ≤ 0.0001 Table 4). Females had slightly lower odds of dabigatran use compared to males (adjusted OR = 0.87, 95% CI 0.79-0.96) in the all-patient analysis but female gender was not a significant predictor in the OAC-naïve analysis. Dabigatran use was also less likely among patients in Midwestern health plans than health plans in the West (all-patient: adjusted OR = 0.55, 95% CI 0.49-0.62; OAC-naïve: adjusted OR = 0.43, 95% CI 0.34-0.56). For both the all-patient and OAC-naïve patient cohorts, pre-index hypertension (all-patient: adjusted OR = 1.16, 95% CI 1.04-1.30; OAC-naïve: adjusted OR = 1.32, 95% CI 1.04-1.69) and ischemic stroke (all-patient: adjusted OR = 1.34, 95% CI 1.18-1.53; OAC-naïve: adjusted OR = 1.42, 95% CI 1.06-1.90) were associated with greater odds of dabigatran use. For both the all-patient and OAC-naïve patient cohorts, pre-index HF and higher risk of bleeding (based on ATRIA score) were associated with lower adjusted odds of dabigatran use. Among the all-patient cohort, prior use of warfarin was strongly associated with lower adjusted odds of dabigatran use (OR = 0.34, 95% CI 0.31-0.38). For the all-patient cohort only, pre-index AF-related procedures, anti-platelet/NSAID medications, dyspepsia medications, rate/rhythm control medications, and pre-index TIA were all positively associated with dabigatran use; odds ratios and associated 95% confidence intervals are shown in Table 4.

**Table 4:** Logistic regression of dabigatran use (versus warfarin) among all patients (n = 20,320) and OAC-naïve patients (n = 2,405)

**Independent variables**	**All-patient cohort†**				**OAC-naïve cohort#**			
	**Odds ratio**	**95% confidence limits**		**P-value**	**Odds ratio**	**95% confidence limits**		**P-value**
Intercept	-1.34			<.0001	-1.26			<.0001
Female (reference = male)	0.87	0.79	0.96	0.0035	1.02	0.83	1.26	0.8362
55-64 age group (reference = 18–54)	1.00	0.86	1.16	0.9517	0.90	0.68	1.21	0.4865
65-74 age group	0.64	0.55	0.75	<.0001	0.59	0.43	0.81	0.0010
75-84 age group	0.44	0.37	0.53	<.0001	0.40	0.28	0.58	<.0001
85+ age group	0.39	0.31	0.48	<.0001	0.42	0.26	0.66	0.0002
Midwest region indicator (reference = west)	0.55	0.49	0.62	<.0001	0.43	0.34	0.56	<.0001
Northeast region indicator	0.90	0.80	1.01	0.0816	0.93	0.70	1.23	0.6125
South region indicator	1.05	0.93	1.19	0.4389	1.12	0.85	1.49	0.4282
PPO insurance indicator (reference = HMO)	1.68	1.48	1.92	<.0001	1.47	1.10	1.96	0.0089
Other insurance type indicator	1.31	1.13	1.52	0.0003	1.20	0.87	1.66	0.2770
Total ATRIA score	0.98	0.95	1.00	0.0477	0.91	0.86	0.96	0.0005
Pre-index heart failure	0.78	0.70	0.87	<.0001	0.76	0.59	0.97	0.0253
Pre-index hypertension	1.16	1.04	1.30	0.0083	1.32	1.04	1.69	0.0246
Pre-index warfarin	0.34	0.31	0.38	<.0001				
Pre-index any anti-platelet/NSAIDS fill	1.15	1.03	1.28	0.0097				
Prior use of any dyspepsia medication	1.13	1.03	1.25	0.0146				
Cardiology specialty of index OAC prescriber indicator (reference = PCP/family/internal medicine)	3.12	2.77	3.51	<.0001	3.59	2.68	4.81	<.0001
Other specialty of index OAC prescriber	1.94	1.72	2.20	<.0001	2.22	1.65	2.97	<.0001
Pre-index PT test use	0.70	0.64	0.78	<.0001				
Pre-index any heart rate/rhythm control medication fill	1.19	1.06	1.34	0.0037				
Pre-index any Afib related procedure	1.53	1.38	1.71	<.0001				
Pre-index ischemic stroke	1.34	1.18	1.53	<.0001	1.42	1.06	1.90	0.0183
Pre-index TIA	1.24	1.02	1.51	0.0281				
Total patient out of pocket pharmacy costs (per $1 K) during the pre-index period	1.04	1.01	1.08	0.0251	1.32	1.17	1.48	<.0001

†C-statistic = 0.7530; Hosmer and Lemeshow Test (p-value = 0.6416).

#C-statistic = 0.7360; Hosmer and Lemeshow Test (p-value = 0.0754).

Health plan characteristics were significantly associated with the odds of dabigatran use, adjusted for other factors (Table 4). Specifically, patients covered by PPO benefit designs were 68% and 47% more likely to be prescribed dabigatran than patients covered by HMO benefit designs in the all patient and OAC-naïve cohorts, respectively. Compared to patients prescribed their index OAC by a primary care/family/internal medicine physician, patients whose index OAC was prescribed by a cardiologist (all-patient: adjusted OR = 3.12, 95% CI 2.77-3.51; OAC-naïve: adjusted OR = 3.59, 95% CI:2.68-4.81) or other specialist (all-patient: adjusted OR = 1.94, 95% CI 1.72-2.20; OAC-naïve: adjusted OR = 2.22, 95% CI:1.65-2.97) were more likely to receive dabigatran. Finally, for both study cohorts, higher total pre-index period patient out-of-pocket pharmacy costs (per $1,000) were associated with greater odds of dabigatran use (all-patient: adjusted OR = 1.04, 95% CI 1.01-1.08; OAC-naïve: adjusted OR = 1.32, 95% CI:1.17-1.48).

## Discussion

Overall, our study found that dabigatran was the oral anticoagulant prescribed for 34% of OAC-naïve patients with NVAF, and that patient demographic and baseline clinical characteristics were related to the use of dabigatran versus warfarin. Consistent with findings of other studies that physicians are less likely to use anticoagulation among older patients, even though older patients have a greater risk of stroke [21-23], this study found that warfarin (as compared to dabigatran) was more likely to be prescribed to older patients. This study also found that patients with a history of HF and those with prior renal function impairment were less likely to use dabigatran. Furthermore, we found that patients who received warfarin therapy previously were less likely to receive dabigatran, and only about 15% of patients who received dabigatran on the index date received warfarin prescriptions after the index date among the all-patient cohort.

Patients with a higher bleeding risk were less likely to use dabigatran. This is an interesting finding because the lack of a monitoring assay and reversal agent for dabigatran therapy have been important safety concerns for clinicians; cases of bleeding events associated with dabigatran, especially in elderly patients with impaired renal function, have been published in the literature and have received recent regulatory attention in the US and elsewhere [24-28]. Compounded with the fact that dabigatran demonstrated an increased risk of intracranial bleeding among patients aged 75 years and older in a phase 3 study, it is not surprising that warfarin use was more frequent among those with a higher risk of bleeding [29]. While the Food and Drug Administration (FDA) is continuing to monitor new sources of drug surveillance data, they have concluded that bleeding rates associated with new dabigatran use do not appear to be greater than those associated with new warfarin use [28].

Cardiologists were significantly more likely to prescribe dabigatran than other physician types. This finding is consistent with prior research [30,31] that found that specialists tend to be early adopters of new drugs. Given that our study used data corresponding to the first year subsequent to dabigatran approval in the US, cardiologists were possibly more familiar with the literature regarding the use of the newly approved dabigatran than other specialists. Indeed, among 65 prescribers surveyed in a study by Huang et al., who were primarily general internists, 63% cited limited experience with dabigatran as a reason for not prescribing dabigatran; compared to cardiologists, general internists were less likely to prescribe dabigatran to OAC-naïve patients due to limited experience with dabigatran (31% versus 69%, p = 0.02) [16]. In addition, health plan factors were associated with the choice of OAC therapy. Particularly, patients covered by PPO benefit designs were more likely to receive dabigatran than patients covered by HMO benefit designsand patients with health plans located in the West region were more likely to receive dabigatran than patients who lived in the Midwest. According to the Kaiser Family Foundation's Employer Health Benefits 2010 Annual Survey, HMO enrollment is significantly higher in the West and Northeast and significantly lower in the South and Midwest [32]. Differences in regional practice variations could be behind the variations in the use of dabigatran. Previous studies have consistently found that increasing copayment amounts lead to a reduction in the use of drugs [33-35]. However, data on how prior out-of-pocket costs predict future medication costs is limited. In our study, patients with higher pre-index out-of-pocket prescription drug costs were more likely to receive dabigatran than warfarin.

Further research is needed to better understand the role of patient income and sociodemographic variables and prior prescription drug expenditures as they relate to patients’ willingness-to-pay for dabigatran. Factors related to OAC therapy choice may change over time as prescribers and patients gain more familiarity and experience with the newer OACs, and additional research will be needed to identify predictors of treatment and changes in OAC treatment patterns in the future. Also, as recommended by the European Society of Cardiology [36] and the American College of Chest Physicians [37], future research needs to consider the impact of patient preferences in OAC therapy decisions. This is important because prior treatment experience and the value that patients place on stroke prevention and bleeding risk affect patients’ willingness to take warfarin [38,39].

### Limitations

Several limitations should be noted in the interpretation of our study’s findings. Although a number of relevant patient factors were analyzed in this study, other patient factors such as patient socioeconomic status, race, and use of over-the-counter medications such as NSAIDs, are not available in administrative claims and therefore were not included. We were not able to assess patient awareness of dabigatran therapy through direct-to-consumer advertising, nor were we able to control for any effect that treatment outcomes (e.g., bleeding) associated with prior warfarin therapy may have had on dabigatran use in our analyses. Nevertheless, the findings of this study were consistent in the all-patient and OAC-naïve cohorts and support the robustness of this study.

## Conclusions

Factors associated with use of newer oral anticoagulant therapy include patient demographic and clinical characteristics, specialty of the healthcare provider, health plan type, and prior prescription out-of-pocket costs. Cardiology specialty of prescribing physician and PPO health plan were the strongest predictors of dabigatran use among all study patients with NVAF, and likelihood of dabigatran use decreased with increasing patient age. Newer studies are warranted to understand prescriber treatment patterns with available OACs. Additional research is needed as more real-world data becomes available to further examine the use of the newer OACs versus warfarin related to the potential impact on the quality of anticoagulation care.

## Competing interests

This research project was funded by Daiichi Sankyo Inc. JJS, AF and HT are employees of HealthCore Inc., an independent outcomes research organization. AA was an employee of HealthCore Inc. at the time of the study. WJK is an employee of Daiichi Sankyo Inc. WSW is an employee of Christiana Care Health System and is a consultant to Daiichi Sankyo, Inc. AA, JJS, AF, HT report no relationship or financial interest with any entity that would pose a conflict of interest with the subject matter of this article.

## Authors’ contributions

AA, JJS, and HT participated in study conception and design. AA, JJS, AF and HT participated in data acquisition, statistical analysis, and data interpretation. JK and AA participated in drafting the first draft of manuscript. All authors have reviewed and approved the final manuscript. All authors have jointly made the decision to submit the manuscript for publication.

## Pre-publication history

The pre-publication history for this paper can be accessed here:

http://www.biomedcentral.com/1472-6963/14/310/prepub
